# Optical frequency domain imaging (OFDI) represents a novel technique for the diagnosis of giant cell arteritis

**DOI:** 10.1038/s41433-024-03216-9

**Published:** 2024-07-16

**Authors:** Laurence Cox, Christopher B. Schulz, James Slaven, Pav Gounder, Sandeep Arunothayaraj, Osama Alsanjari, James Cockburn, David A. Wright, Huw Oliphant, Saul Rajak

**Affiliations:** 1 King’s College Hospital NHS Foundation Trust, Queen Mary’s Hospital, Frognal Avenue, DA14 6LT London, UK; 2 Sussex Eye Hospital, University Hospitals Sussex NHS Foundation Trust, Eastern Road, BN2 5BF Brighton, UK; 3 Brighton and Sussex Medical School, University of Sussex, BN1 9PX Brighton, UK; 4 Sir Charles Gairdner Hospital, Hospital Avenue, Nedlands, Perth, WA 6009 Australia; 5 Centre for Ophthalmology and Visual Science, University of Western Australia, Perth, WA Australia; 6 Department of Cardiology, University Hospitals Sussex NHS Foundation Trust, Eastern Road, BN2 5BF Brighton, UK; 7 Department of Histopathology, University Hospitals Sussex NHS Foundation Trust, Eastern Road, BN2 5BF Brighton, UK

**Keywords:** Predictive markers, Optical spectroscopy

## Abstract

**Background/Objectives:**

Giant cell arteritis (GCA) is an inflammatory vascular disease in which prompt and accurate diagnosis is critical. The efficacy of temporal artery biopsy (TAB) is limited by ‘skip’ lesions and a delay in histological analysis. This first-in-man ex-vivo study aims to assess the accuracy of optical frequency domain imaging (OFDI) in diagnosing GCA.

**Subjects/Methods:**

29 TAB samples of patients with suspected GCA were submerged in 0.9% sodium chloride and an OFDI catheter was passed through the lumen to create cross-sectional images prior to histological analysis. The specimens were then preserved in formalin for histological examination. Mean intimal thickness (MIT) on OFDI was measured, and the presence of both multinucleate giant cells (MNGCs) and fragmentation of the internal elastic lamina (FIEL) was assessed and compared with histology, used as the diagnostic gold standard.

**Results:**

MIT in patients with/without histological evidence of GCA was 0.425 mm (±0.43) and 0.13 mm (±0.06) respectively compared with 0.215 mm (±0.09) and 0.135 mm (±0.07) on OFDI. MIT measured by OFDI was significantly higher in patients with histologically diagnosed arteritis compared to those without (*p* = 0.0195). For detecting FIEL and MNGCs, OFDI had a sensitivity of 75% and 28.6% and a specificity of 100% and 77.3% respectively. Applying diagnostic criteria of MIT > 0.20 mm, or the presence of MNGCs or FIEL, the sensitivity of detecting histological arteritis using OFDI was 91.4% and the specificity 94.1%.

**Conclusions:**

OFDI provided rapid imaging of TAB specimens achieving a diagnostic accuracy comparable to histological examination. In-vivo imaging may allow imaging of a longer arterial section.

## Introduction

Giant cell arteritis (GCA) is a systemic inflammatory vascular disease which carries a risk of blindness, stroke, and aortic aneurysm if untreated. The incidence rises with age to 70/100,000 in women aged 70–79 years [1]. The usual treatment includes a prolonged course of high-dose corticosteroids and, if GCA is suspected, treatment should not be delayed while diagnostic confirmation is awaited [2]. Owing to the risk of both disease-associated and treatment-associated morbidity and mortality, prompt and accurate diagnosis is critical. Current diagnostic modalities include temporal artery biopsy (TAB), ultrasound, magnetic resonance imaging (MRI), and ^18^F-fluorodeoxyglucose positron emission tomography/computed tomography (^18^F-FDG PET/CT). TAB is by far the most widely used investigation, however, it has significant limitations including a relatively low sensitivity of 77% (95% confidence interval: 71.8–81.9%) [3], delays whilst awaiting histological reporting, and surgical complications.

Optical frequency domain imaging (OFDI) may represent a promising alternative diagnostic modality. The technology is already widely used in clinical ophthalmology (as ocular coherence tomography (OCT) for imaging the retina, optic disc and anterior segment) and in cardiology for intravascular imaging [4, 5]. In vascular OFDI, an infrared probe is passed into the lumen of a vessel and, using second-generation OCT, provides cross-sectional imaging of the vessel walls with a resolution of 10–20 microns. In comparison to non-invasive diagnostic modalities, such as ultrasound, PET/CT and MRI, this potentially offers a significant improvement in resolution, whilst in comparison to TAB it provides immediate bedside results and allows a longer section of the artery to be examined which may improve diagnostic sensitivity.

This first-in-man ex-vivo study aims to assess the clinical efficacy of OFDI in aiding the diagnosis of GCA in the acute setting. The primary outcome of this study was the diagnostic accuracy (sensitivity and specificity) of OFDI in detecting histologically confirmed arteritis. Secondary outcomes included evaluating the accuracy of several OFDI-derived proxy markers for a histological diagnosis of GCA. These markers included measuring mean intimal wall thickness (MIT), detecting multinucleate giant cells (MNGC), and detecting fragmentation of the internal elastic lamina (FIEL).

## Methods

Consecutive patients undergoing TAB for suspected GCA in a single University Teaching Hospital in the United Kingdom were invited to participate in the study from November 2019 to August 2022 until an adequate sample size was achieved for reasonable statistical power.

Ethical approval was granted by London – City & East Research Ethics Committee with REC reference number 19/LO/1117. Patients were consented for all study procedures, and the study was conducted in accordance with the guidelines of the declaration of Helsinki.

### Study procedures

TAB was undertaken using standard technique to excise an approximately 2 cm portion of the superficial temporal artery. A 7-0 polyglactin 910 (Vicryl) suture loop was placed at each end of the specimen. The artery sample was submerged in 0.9% sodium chloride and pinned to a board under slight tension using the suture loops at either end.

The *Terumo (Tokyo, Japan) FastView Coronary Imaging* OFDI catheter (item reference number: OP-16P2613)) was passed through the lumen of the biopsy specimen and set at a pullback speed of 5 mm/second. The OFDI imaging system (*Terumo Lunawave Coronary Optical Coherence Tomography Imaging System* with *Lunawave Version 1.2 Software* (item reference number: LW*10SG3) thereby created a continuous cross-sectional image of the wall of the biopsy specimen. The sample was then immediately transferred to 10% formaldehyde and transported to the histopathology laboratory.

### OFDI assessment

**Fig. 1:**
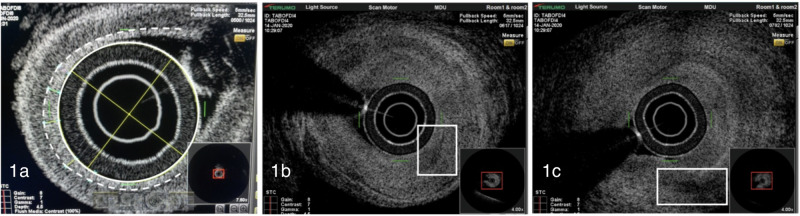
Cross sectional images of temporal artery biopsy specimens created by optical frequency domain imaging. **a** Image showing the measurement of intimal wall thickness from the lumen (solid line) to the outer extent of the intima (dashed line). **b** Image showing possible FIEL (white square). **c** Image showing the possible presence of MNGC/lymphocytic infiltrate (white square).

The intimal thickness was measured at three distinct points along its length. All OFDI images were interpreted using the *Lunawave Version 1.2 Software*, by the same investigator, using a standardised method for measuring intimal thickness. The points at which the intimal thickness was measured equated approximately to the start, middle and end of the specimen length dependent on where the intima could clearly be visualised and easily measured. The intima was visualised as a distinct area of hyperreflectivity, and measurements were taken radially from the inner wall to the outer most limit of the intima (Fig. 1a). MIT was calculated from the three measurements taken. The images were also assessed for evidence of FIEL and MNGCs (Fig. 1b/c). To reduce observer bias, the images were interpreted without access to histological findings or clinical history.

### Histopathological assessment

Following fixation in formalin, automated tissue processing and embedding in paraffin, 3 µm sections were stained with haematoxylin and eosin (H&E) and Elastin van Geison (EVG). Intimal thickness was determined to be the shortest distance from the internal elastic lamina to the inner most extent of the intima and was measured using the microscope Vernier scale. Each specimen was then examined for the presence or absence of transmural inflammation, MNGCs, and FIEL. In the absence of evidence of inflammation, further H&E sections were examined until the tissue had been exhausted. Examination was performed by a single experienced histopathologist who then determined whether the features were consistent with GCA. The histopathologist had access to the clinical history on the histopathology request form but did not have access to the OFDI measurements.

### Statistical analysis

Statistical analysis was performed using *R* [6]. Sensitivity and specificity of OFDI to detect histological presence of MNGCs and FIEL was calculated. The agreement between MIT measured by OFDI and that measured histologically was evaluated according to the Bland–Altman method. As the two sets of data were normally distributed, a two-tailed unpaired t-test was used to ascertain the statistical significance of the difference in MIT as measured by OFDI between patients with histological evidence of arteritis and those with no histological evidence of arteritis. A *P* value of <0.05 was interpreted as statistically significant. A receiving operator curve (ROC) was plotted to evaluate the diagnostic ability of OFDI-derived MIT in detecting histologically confirmed arteritis and to derive an optimal cut-off point for diagnostic utility. This cut-off point was used in combination with the presence of OFDI-observed MNGC or FIEL to provide a final OFDI-based diagnostic criteria. The sensitivity and specificity of this criteria in detecting histological-confirmed arteritis was calculated.

## Results

30 patients were enrolled in the study. One patient’s biopsy specimen was lost in transit to the histopathology lab, with no available histology, the sample was excluded from the analysis. Otherwise, no TAB samples were damaged as a result of the OFDI procedure, and there was no morbidity or mortality as a result of the study. Of the 29 included patients, 17 (58.6%) were male. The mean (±standard deviation) age was 74.9 (±9.7) years. The mean (±standard deviation) time between starting steroids and undergoing TAB was 7.6 (±5.6) days. The mean (±standard deviation) length of TAB specimen after formalin fixation was 16 (±3.4) mm. Histologically diagnosed arteritis was confirmed in 12 (40%) patients based on the presence of transmural inflammation and FIEL with or without the presence of MNGC (Table 1).

**Table 1:** Individual-level data with histological findings on biopsy and optical frequency domain imaging (OFDI).

Histological findings									Findings on optical frequency domain imaging			
Case	Days between starting systemic steroids and biopsy	Biopsy specimen length (cm)	Mean Intimal Thickness (mm)	Fragmentation of internal elastic lamina	Transmural inflammation	Giant cells	Diagnosed with GCA		Mean intimal thickness (mm)	Fragmentation of internal elastic lamina	Giant cells	Mean intimal thickness > 0.2 mm OR fragmentation of internal elastic lamina OR giant cells
4	5	21	1.25	+	+	+	+		0.297	+	−	+
8	2	20	0.3	+	+	−	+		0.087	−	−	−
9	7	13	0.2	+	+	+	+		0.11	+	−	+
14	7	18	0.1	+	+	−	+		0.167	−	+	+
16	4	16	0.1	+	+	−	+		0.307	+	+	+
19	3	12	1.3	+	+	+	+		0.277	+	+	+
20	2	20	0.2	+	+	+	+		0.17	+	−	+
22	2	13	0.2	+	+	+	+		0.237	−	−	+
24	2	10	0.1	+	+	+	+		0.1	+	−	+
25	2	15	0.7	+	+	−	+		0.207	+	+	+
26	11	12	0.45	+	+	+	+		0.223	+	+	+
27	11	20	0.2	+	+	−	+		0.393	+	+	+
1	6	18	0.1	−	−	−	−		0.113	−	−	−
2	0	17	0.1	−	−	−	−		0.11	−	−	−
3	9	16	0.2	−	−	−	−		0.147	−	−	−
5	8	15	0.3	−	−	−	−		0.157	−	−	−
6	6	14	0.1	−	−	−	−		0.083	−	−	−
7	7	16	0.1	−	−	−	−		0.077	−	−	−
10	10	15	0.1	−	−	−	−		0.2	−	−	−
11	7	22	0.1	−	−	−	−		0.177	−	−	−
12	10	18	0.1	−	−	−	−		0.063	−	−	−
13	8	17	0.1	−	−	−	−		0.17	−	−	−
15	0^a^	21	0.1	−	−	−	−		0.083	−	−	−
17	20	11	0.1	−	−	−	−		0.103	−	−	−
18	9	18	0.1	−	−	−	−		0.09	−	−	−
21	8^a^	12	0.1	−	−	−	−		0.333	−	+	+
28	11	20	0.1	−	−	−	−		0.137	−	−	−
29	26	11	0.2	−	−	−	−		0.11	−	−	−
30	9	15	0.2	−	−	−	−		0.137	−	−	−

^a^Denotes a patient on long-term low dose (2–5 mg per day) oral prednisolone.

MIT (±standard deviation) among patients with no histological evidence of GCA was 0.129 mm (±0.061) on histology and 0.135 mm (±0.064) on OFDI. MIT (±standard deviation) on histology among arteritis patients was 0.425 mm (±0.432) and on OFDI was 0.215 mm (±0.094). MIT as measured by OFDI was significantly higher in those patients with histologically diagnosed arteritis than those without (*p* = 0.0195).

For detecting histologically confirmed FIEL, OFDI had a sensitivity of 75% and a specificity of 100%. For detecting histologically confirmed MNGC on biopsy specimens, OFDI had a sensitivity of 28.6% and a specificity of 77.3%.

**Fig. 2:**
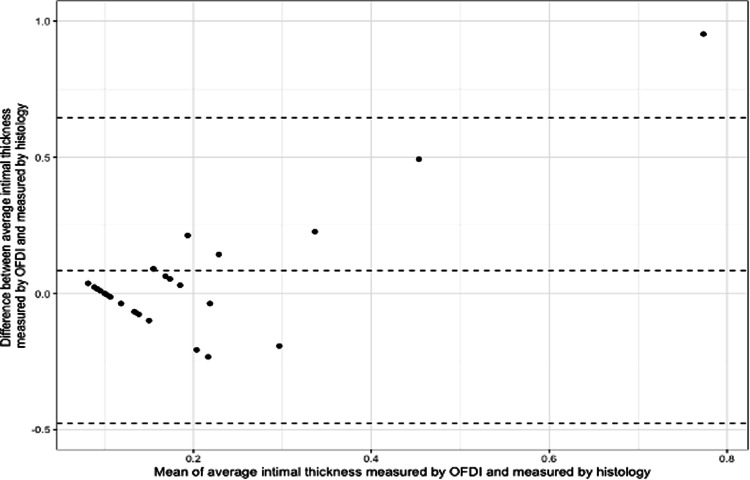
Bland–Altman plot comparing the mean values and difference between values for mean intimal thickness measured by Optical Frequency Domain Imaging (OFDI) and by histology. Dashed lines represent the mean difference along with upper and lower 95% levels of agreement.

Figure 2 shows the agreement in MIT measured by both OFDI and histology according to the Bland–Altman method. Measurements by OFDI were on average 0.08 mm higher than those acquired histologically. The 95% levels of agreement were −0.48 mm and 0.65 mm.

**Fig. 3:**
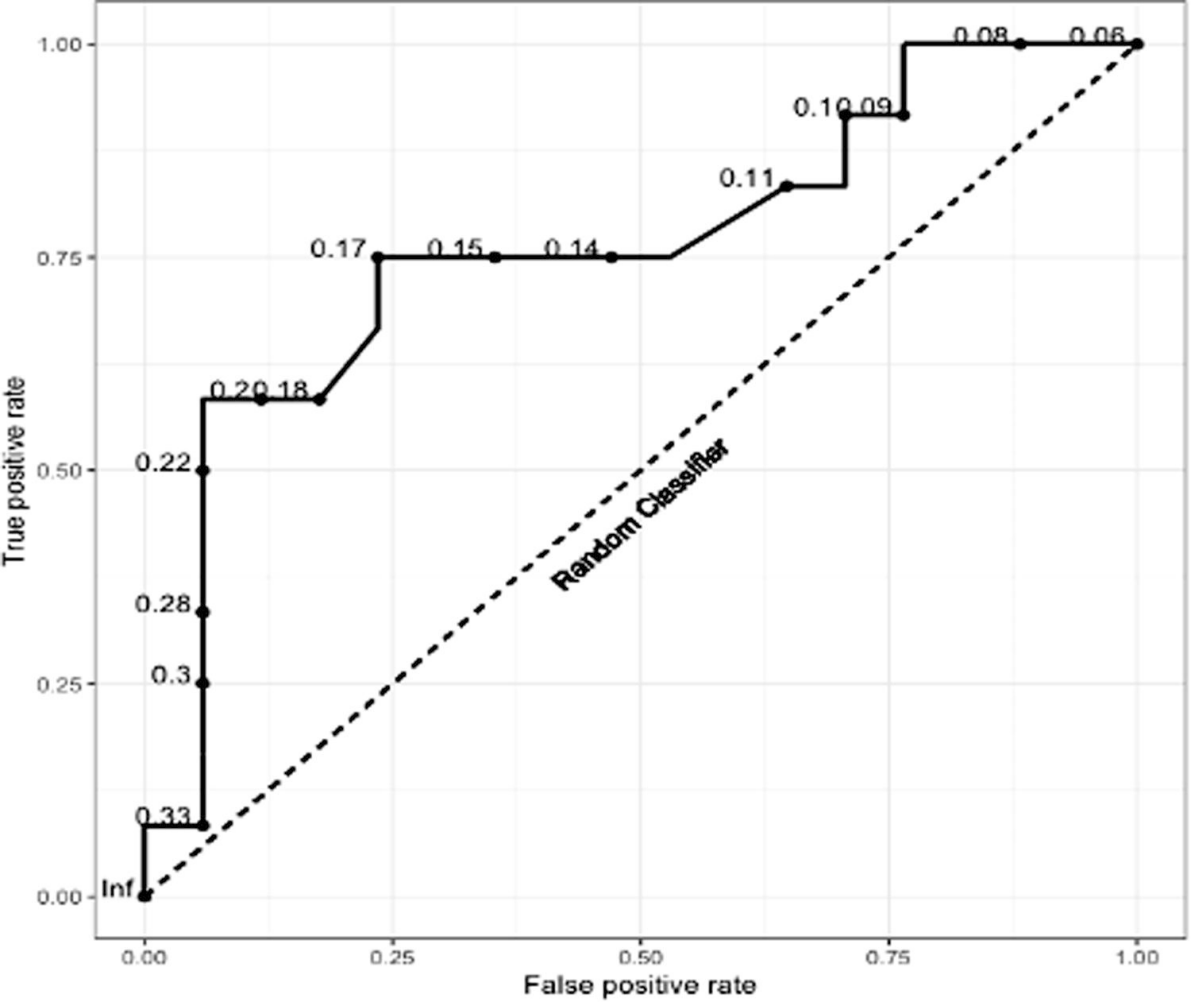
Receiver Operating Curve (ROC) for diagnosing histological arteritis using mean intimal thickness measured by optical frequency domain imaging. Dashed line represents the ROC of a random (chance) classifier.

Figure 3 shows the receiver operating characteristic (ROC) curve for variable cut off values of MIT as measured by OFDI, at or above which, the patient is diagnosed with giant cell arteritis. Based on the ROC curve, a cut-off point of 0.20 mm is the optimum for maximising specificity (94.1%) without compromising sensitivity to below 50% (sensitivity = 58.3%). A cut-point of 0.17 mm maximises sensitivity (76.4%) without compromising specificity to below 50% (specificity = 66.7%). When each of these threshold values for MIT (measured by OFDI) are considered along with the presence of either MNGCs or FIEL seen on OFDI, the ability to detect arteritis was further enhanced (Table 2). Using the diagnostic criteria of MIT > 0.20 mm, and the presence of MNGCs or FIEL, the sensitivity of detecting histological arteritis was 91.4% and the specificity 94.1%. Only one patient was incorrectly diagnosed with arteritis by OFDI. This patient had been on long-term corticosteroids for seronegative arthritis and despite negative biopsy continued to be treated as clinically possible GCA at the most recent clinical follow up after 12 months.

**Table 2:** Diagnostic accuracy (sensitivity and specificity) of optical domain frequency imaging (OFDI) to detect histologically confirmed arteritis using two different criteria.

	Findings on optical domain frequency imaging (OFDI) of temporal artery biopsy specimen	
	**Mean intimal thickness** > **0.17** **mm**	**Mean intimal thickness** > **0.20** **mm**
	**OR**	**OR**
	**fragmentation of internal elastic lamina**	**fragmentation of internal elastic lamina**
	**OR**	**OR**
	**giant cells**	**giant cells**
Sensitivity to detect histologically confirmed arteritis	91.70%	91. 7%
Specificity to detect histologically confirmed arteritis	76.50%	94.10%

Sensitivity and specificity achieved by OFDI in detecting histologically diagnosed arteritis at varying cut-off MIT values.

## Discussion

This first in man real-world prospective study investigated the use of ex vivo OFDI for the diagnosis of GCA in the acute setting. In this study, OFDI was able to provide rapid imaging of TAB specimens with a diagnostic accuracy approaching that of histological examination.

A range of diagnostic techniques are used in acute GCA, but each has drawbacks and limitations. There is no single test that can be relied upon for the diagnosis of GCA and tests must be interpreted within the context of the clinical history, examination, and laboratory features including erythrocyte sedimentation rate, C-reactive protein, and platelet count. While TAB is still considered the gold standard, owing to its specificity of 100% [7], it has significant shortcomings [1]. The estimated sensitivity is 77% [3], due to a false negative rate of up to 44% [8] which likely stems either from the 1–2 cm biopsy specimen being within a ‘skip lesion’ – a part of the vessel that does not contain histopathological features of the disease, or from iatrogenic damage to the biopsy specimen [2]. Furthermore, the delay in histopathological examination and reporting may result in a period of potentially unnecessary high-dose corticosteroid treatment. TAB also carries a risk of surgical complications such as bleeding, infection and facial nerve injury [4] which may be greater than previously assumed [7]. Non-invasive methods of GCA diagnosis such as ultrasound, T1 weight contrast-enhanced MRI diagnosis and ^18^F-FDG PET/CT negate these risks but carry limitations of their own. Ultrasound achieves a sensitivity of 68–88% and a specificity of 77–96% [9–11], and although the specificity of ultrasound is greatly enhanced in the presence of a halo sign [12–14], only 63% of patients diagnosed with GCA on TAB have a halo sign on ultrasound [15]. Recent improvement in ultrasound technology and higher-resolution imaging have led to high inter-rater and intra-rater agreement with optimism for improved utility in future [16]. The modality still requires high levels of expertise and experience. Other less commonly utilised modalities include T1 weighted contrast-enhanced MRI with a sensitivity of 78–94% and specificity of 78–90% [17–19], and ^18^F-FDG PET/CT with a sensitivity of 64–92% and a specificity of 85–100% [20–22]. However, MRI is expensive, time consuming, and contraindicated in certain patients such as those with magnetic prostheses, while ^18^F-FDG PET/CT carries the long-term risks associated with low-level radiation exposure [23, 24], specifically an increased risk of genetic aberrations and neoplastic disease. Both modalities may be less widely available for immediate diagnostic purposes, particularly in smaller centres and during unsocial working hours.

OFDI has several important advantages over other diagnostic modalities. As a partially invasive investigation, in-vivo OFDI would likely present a lesser risk of procedural complications than TAB. The use of catheter angiography is well established within cardiology and carries less than a 1% risk of significant complications [25]. Owing to an image resolution (10–20 μm) that is far superior to any non-invasive diagnostic techniques, it has been shown that OFDI can detect cellular level changes such as FIEL and the presence of MNGCs. This may be particularly beneficial in defining early pathological change, or subtle diagnostic cellular pathology in patients on corticosteroids. Furthermore, with results that are rapidly available without the need for histological processing and evaluation, OFDI is less likely to delay diagnosis and the associated risks of high-dose corticosteroid treatment.

Intimal wall thickening is a useful and important clinical parameter that, as this study has shown, can be detected on OFDI, aiding the diagnosis of acute vasculitis. Although histological intimal wall thickness will vary over the specimen length due to ‘skip lesions’, it is considered an objective measurement and a defining feature of acute vasculitis. Marked histopathological thickening of the intima is strongly associated with increased inflammatory infiltrate [26] and the extent of intimal thickening seems to be predictive of the development of ischaemic neuropathy and ophthalmic symptoms [27]. Additionally, it has previously been shown that an increased intima-media thickness can be used to distinguish vasculitic from normal arteries in suspected GCA [28] and current evidence suggests that high-resolution imaging of intimal thickness is a robust measurement indicative of vasculitis that is minimally effected by corticosteroid use [29]. This study yielded a difference in MIT measured by OFDI and histology. This may derive from differences in preparation, with OFDI being conducted on specimens submerged in saline and histology on specimens prepared in formalin, but also from differences in precisely which section of the intima was being measured in the two methods. Moreover, the tissue penetration of the OFDI technology used in the present study allows for the measurement of a maximum wall thickness of approximately 0.4 mm. This is sufficient to confirm the presence of increased intimal thickness but cannot provide a precise value for the thickest intimas. Greater tissue penetration may be achievable with longer wavelength light, but this would be at the compromise of image resolution.

MNGCs are present in approximately 50% of temporal artery biopsies and within this context, are considered pathognomonic of GCA [30]. The diameter of MNGCs is highly variable but typically ranges from 40–120 um [31]. Therefore, the resolution of OFDI is theoretically sufficient for MNGCs to be visible. As such, it is not clear why the sensitivity for the detection of MNGCs was so low when compared with TAB. It may be a result of inadequate user experience, a lack of chromatic contrast between MNGCs and the surrounding tissue, or artifacts degrading the quality of the images. Image artifacts are largely avoidable but can be caused by inadequate blood flushing the vessel, air in the imaging catheter sheath, or eccentric wire positioning [32]. With greater user experience, simple post-imaging modification such as brightness or contrast adjustment, and the minimisation of image artifacts, it may be possible to improve the sensitivity of OFDI for detecting MNGCs. Alternatively, with sufficient raw data, automated image analysis (using edge detection and/or segmentation and the subsequent construction of recurrent neural networks) may achieve greater sensitivity than human interpretation of images.

Although FIEL is a common finding in GCA [33], as an isolated finding, it is of limited diagnostic significance owing to its low specificity and common presence as part of a normal aging process [34]. However, the high sensitivity that OFDI achieved in detecting FIEL is encouraging. Despite its low clinical specificity, it does represent a useful supplementary finding; when used in combination with other OFDI-derived measures (MIT > 0.20 mm and the presence of MNGCs) a high sensitivity (91.7%) and specificity (94.1%) in detecting histologically confirmed arteritis was achieved.

As with any novel imaging modality, observer skill takes time to develop. Despite this, the sensitivity and specificity that OFDI achieved in this study are promising. This sensitivity and specificity was achieved with the 16 mm segments of artery that were excised for histopathological examination and will therefore be subject to the same risk of skip lesions as TAB. Specimen length is itself known to be an independent prognostic factor for a positive TAB result and it is anticipated that ‘on table’ OFDI would be able to image a longer section of the artery [35]. The diameter of the *Fastview Coronary Imaging Catheter* is 0.87 mm, and therefore should be able to image the entire 2.5 to 5 cm length of the superficial temporal artery even if the lumen is narrowed by arteritic change [36, 37].

OFDI is not without shortcomings. In severely inflamed and narrowed temporal arteries, catheterisation may be very challenging. It is possible that TAB would still be required in such scenarios, though biopsy would likely have a high sensitivity in such instances. Furthermore, although OFDI images and results are instantaneously available, a delay in identifying suitable operating space and OFDI expertise may still cause delay. OFDI units are expensive, and the probes are single use and costly. However, the required equipment is increasingly available in cardiology units, and it is feasible that GCA OFDI could be done in the cardiac catheterisation laboratory.

Limitations of this study primarily pertain to the use of TAB as a gold standard investigation and the direct comparison with OFDI images. To prevent damage or disruption of the TAB specimens, OFDI had to be performed promptly after the biopsy was taken. The speed with which this was performed likely compromised the quality of the OFDI images. Although this study made a direct comparison between the parameters of fresh ex-vivo tissue (OFDI) with formalin-fixed tissue (TAB), it is not clear whether the intimal wall thickness remains constant in thickness and composition during the fixation process. Lastly, the intimal wall thickness on OFDI was calculated as a mean thickness along the vessel length, measured at points where the intima was most clearly visible and defined, whereas the intimal wall thickness on histology was measured at its thinnest point.

In conclusion, this study represents the first use of intravascular ex-vivo OFDI in the diagnosis of GCA. This proof-of-concept study demonstrates both feasibility and promising diagnostic potential. The next steps in the development of this technique involve testing the reproducibility of results across different users and testing the utility of OFDI in vivo.

## Summary

### What was known before

- Giant cell arteritis is an inflammatory vascular disease that carries a risk of stroke, blindness and aortic aneurysm if not promptly treated.
- Giant cell arteritis is notoriously challenging to diagnose.
- Temporal artery biopsy is the most commonly used method for diagnosing giant cell arteritis but has significant drawbacks including a low sensitivity and the risk of surgical complications.

### What this study adds

- This study introduces optical frequency domain imaging as a potential diagnostic technique for diagnosing acute giant cell arteritis.
- OFDI was able to detect histologically confirmed giant cell arteritis with promising sensitivity and specificity.

## Supplementary information

Consort Checklist

Eye Reporting Checklist

## Data Availability

The authors of this manuscript declare that the materials used in the manuscript, including all raw data, will be freely available to any researcher wishing to use them for non-commercial purposes, without breaching participant confidentiality.
